# Enhancing the defensibility of examiners’ marks in high stake OSCEs

**DOI:** 10.1186/s12909-017-1112-z

**Published:** 2018-01-06

**Authors:** Boaz Shulruf, Arvin Damodaran, Phil Jones, Sean Kennedy, George Mangos, Anthony J. O’Sullivan, Joel Rhee, Silas Taylor, Gary Velan, Peter Harris

**Affiliations:** Faculty of Medicine, UNSW, Sydney, Australia

## Abstract

**Background:**

Most assessments in health professions education consist of knowledge-based examinations as well as practical and clinical examinations. Among the most challenging aspects of clinical assessments is decision making related to borderline grades assigned by examiners. Borderline grades are commonly used by examiners when they do not have sufficient information to make clear pass/fail decisions. The interpretation of these borderline grades is rarely discussed in the literature. This study reports the application of the Objective Borderline Method (version 2, henceforth: OBM2) to a high stakes Objective Structured Clinical Examination undertaken at the end of the final year of a Medicine program in Australia.

**Methods:**

The OBM2 uses all examination data to reclassify borderline grades as either pass or fail. Factor analysis was used to estimate the suitability of data for application of OBM2. Student’s t-tests, utilising bootstrapping, were used to compare the OBM2 with ‘traditional’ results. Interclass correlations were used to estimate the association between the grade reclassification and all other grades in this examination.

**Results:**

The correlations between scores for each station and pass/fail outcomes increased significantly after the mark reclassification, yet the reclassification did not significantly impact on students’ total scores. Examiners, students and program leaders expressed high levels of satisfaction and the Faculty’s Curriculum Development Committee has decided that the OBM2 will be used for all future clinical examinations. Implications of the OBM2 are discussed.

**Conclusions:**

The OBM2 provides a feasible, defensible and acceptable solution for classification of borderline grades as either pass or fail.

## Background

For clinical skills assessment in health professions education, it is commonly believed that examiners apply their best judgement when providing feedback on student performance in Objective Structured Clinical Examinations (OSCE) [1]. However, do we know how good examiners’ judgements are, considering they are required to grade students on a number of criteria in a short time period? Previous studies suggest that examiners felt less confident when giving a fail grade than when giving a pass grade [2, 3]. Influences such as examiners’ familiarity with the examinees [4], examinees’ first impression on examiners [5], and other biases such as gender and culture, may impact on examiners’ judgements [6]. Moreover, a comprehensive meta-analysis suggested that OSCE ‘*does not guarantee reliable scores and accurate decisions about medical student*s’ [7] with an overall low calculated reliability (mean un-weighted α = .62 & G = .49), although Brannick and colleagues [7] found that reliability was higher for clinical than for communication skills items.

More recent studies identify other challenges in OSCEs. For example, results from a study on an OSCE used for Exercise Physiology found that the examiners accounted for 24.1% of the variance in technical skills scores, whereas students accounted only for 4.9% of the variance [8]. Hope and Cameron [9] found that examiners were more lenient at the beginning compared to the end of OSCE examinations. A recent study found that changing examiners at a station during the United Kingdom postgraduate surgery OSCEs made a significant difference to students’ scores, although the reliability of the OSCE did not change [10].

Thus, substantial evidence suggests that examiners’ biases are unavoidable when OSCEs are employed. These biases might not have a major impact when a student’s performance is a clear pass or clear fail. However, when the examiner is unsure or does not have enough evidence to confidently decide whether the student has passed or failed (i.e. performing at a borderline level), the examiner’s biases may play a significant role in determining the pass/fail decision. The literature provides a numerous methods for setting cut-scores for the entire OSCE or for individual stations, of which the most popular are the Borderline Regression Method, the Borderline Groups Method and the Contrasting Groups Method [11–19]. The authors of the AMEE guide no. 49 ‘How to measure the quality of the OSCE: A review of metrics’ favour the Borderline Regression Method (BLR) since it “*uses all the assessment interactions between assessors and candidates, and these interactions are ‘real*” [20] and is “*objectively based on predetermined criteria, using a large number of assessors and generates a wide range of metrics*” [20].

Guided by the principles suggested by Pell and colleagues [20] a new method (The Objective Borderline Method, henceforth: OBM) was introduced to address challenges raised by borderline marks [21–24]. The OBM uses all assessment interactions between assessors and candidates to determine whether a borderline grade should be reclassified as pass or fail and, when applicable, the OBM can be used for determining cut-scores for the entire examination [21]. The OBM utilises predetermined criteria that have been established by all relevant stakeholders as acceptable for determining the level of competency in a particular examination (in this case, OSCE). This study describes the application of a revised version of the OBM, known as OBM2 [23, 24], to a high stakes OSCE undertaken at the end of the final year of the Medicine program at UNSW Medicine, Sydney, Australia.

### Context

The UNSW Medicine program is a six-year undergraduate entry program [25]. This modular program consists of three phases, each of two years. Students undertake examinations throughout the courses, and major barrier examinations are held at the end of each phase. At the end of Phase 3 (year 6) the integrated clinical examination consist of written, structured oral (management viva) and clinical skills examinations [26]. Prior to the implementation of the OBM2, the marking schedule for OSCE items consisted of four categorical grades: Fail (F); Borderline Pass (P-); Clear Pass (P); and Exceeded Expectations/Distinction (P+). To calculate a final result each grade was converted to a numeric score as follows: (F) = 3; (P-) = 5; (P) = 7; (P+) = 9 (out of 10). Students who received P+ in all assessment criteria within a station could have their P+ marks upgraded from 9 to 10 if the examiner believed their performance was outstanding across the board (for details see: [27]).

There were two principal concerns with the existing system. Course and program leaders had expressed that examiners in clinical examinations were too lenient and were reluctant to fail students, thus tending to award P- (i.e Borderline Pass) rather than F grades despite written comments suggesting the student was not at a “pass” level. Similar concerns have been reported elsewhere [9, 28]. The second reason was related to the nature of the P- grade. This was described as a ‘Borderline Pass’, and some examiners perceived it as a ‘conditional pass’. Under the previous marking method two P- grades and no F grades at a station was considered to be an overall pass, but a student with three P- grades failed the station (for details see: [27]). This had a logical flaw since the P- or Borderline Pass grade was neither numerically (converted to 50%) nor descriptively defined as a Fail.

The implementation of the OBM2 [23, 24] aimed to address these concerns. First, the Borderline Pass grade was replaced with a ‘Borderline’ grade, which indicates that the examiner is ‘*unable to decide on whether the student performance was a clear pass or a clear fail related to a particular assessment criterion* (item)*’*. Using a Borderline grade allowed examiners to give an undetermined pass/fail when that was appropriate, and averted the possibility of examiners being forced to make a clear decision when that was not possible. Allowing examiners to give an undetermined ‘Borderline’ grade (B) was designed to reduce the impact of the examiner’s bias on their marking [4, 9], as well as reducing examiner anxiety in difficult cases.

The preparations for the implementation of the OBM2, including changes to the assessment guides and examiner training were carried out throughout the 2016 academic year and the OSCE took place in September 2016. Four relevant Faculty committees independently discussed and approved the process. Student representatives on those committees strongly supported the implementation of the OBM2. A contingency plan was in place if the implementation of the OBM2 was unsuccessful. The OBM2 was fully implemented across all clinical examinations in the UNSW Medicine program in 2016.

The current study focuses on the implementation of OBM2 [23, 24] in the final clinical skills examination (OSCE) undertaken at the end of the Medicine program. The next section describes the OBM and OBM2 in detail.

### The objective borderline method (OBM and OBM2)

#### OBM

The OBM [21] is a standard setting method that produces an overall examination cut-score. The OBM yields an index from two independent proportions of examination grades when the possible grades are classified as: clear pass and above (P); clear fail (F); and borderline (B), which describes an indeterminate grade, i.e. there is insufficient information in the student’s response (examination grade) to determine whether they clearly passed or failed the examination. The two proportions are: (1) the proportion of P grades among all the non-F grades; and (2) the proportion of B grades among all the non-P grades.

If the number of P grades is p; the number of F grades is f; and the number of B grades is b then:

The proportion of the B grades among all the non-P grades is: $\mathsf{\Pr}\left(\mathsf{B}\right)=\mathsf{b}/\left(\mathsf{f}+\mathsf{b}\right)$.

The proportion of the P grades among all the non-F grades is: $\mathsf{\Pr}\left(\mathsf{P}\right)=\mathsf{p}/\left(\mathsf{b}+\mathsf{p}\right)$.

The OBM index is the multiplication of these two proportions: OBM index = Pr(P) × Pr(B) = [p/(b + p)] × [b/(f + b)]. The OBM index, therefore, summarises two levels of difficulty: The difficulty of not getting an F grade (i.e. getting a B grade) given a P mark is not achievable; and the difficulty of getting a P grade given all grades are above clear fail (>F grade). Multiplication of proportions is an acceptable practice for yielding indices derived from observations [29].

Note that although Pr(P) and Pr(B) may relate to each other, they are sufficiently independent since a particular *proportion* of P grades among the P and the B grades *cannot* determine the *proportion* of the B grades among the B and the F grades (and vice versa). The OBM is not applicable when there are no B grades, since no decision is required. *The OBM is also applicable for examination marks on a continuous scale* when there is uncertainty where the cut-score separating passes from fail should be. Thus, to apply the OBM there is a need to determine the minimum score for clear pass and the maximum score for clear fail, whereas the scores that are neither clear pass nor clear fail are defined as borderline. Since the OBM is a multiplication of two proportions, where each is a proportion of a sub-group within a group (i.e Pr(B) = b/(f + b); Pr(P) = p/(b + p)), the OBM index is always ≤1. The OBM index is used to determine the proportion of borderline grades that should be re-classified to Pass; and (1-OBM index) determined the proportion of borderline grades that should be reclassified to Fail [21]. From this classification a cut-score could be estimated (the lowest borderline grade that was reclassified to Pass). It has been previously demonstrated that the cut-scores generated by the OBM were highly correlated with cut-scores generated by other methods [21]. The validity of the OBM was demonstrated utilising an advanced version of the OBM [22]. On average, the accuracy (% classification correct) of the pass/fail decisions made by the OBM was approximately 70%, which is equivalent to an effect size of 1 [30].

#### OBM2

The challenge that neither the OBM nor any other existing standard setting methods could address was related to the *nature of the borderline grade itself*. Before the OBM2 was introduced [23], no other method was available to estimate whether an individual borderline grade should be classified as either pass or fail. It was always assumed that a borderline grade is situated in the middle between Pass and Fail [31, 32], but empirical evidence was never presented to substantiate that assumption.

The introduction of the OBM2 [23, 24] aimed to resolve the uncertainty of the borderline grade, i.e. to determine whether a borderline grade given by an OSCE examiner is more likely to represent a Pass or a Fail performance. The OBM2 is not a standard setting method in the traditional sense, i.e. it does not determine a cut-score on a continuous scale. Nor does OBM2 overwrite the performance criteria set by examiners or item writers. Rather, the OBM2 is a decision making mechanism that aims to resolve examiners’ uncertainty when assessing examinees’ performance. A recent study [24] demonstrated that the OBM2 yielded 77% accuracy, which is equivalent to an effect size of 1.4 [30]. Previous studies using OBM2 utilised data that were not purposely designed for OBM2, i.e. they used data that either retrospectively determined a borderline range for overall score [24], or data that used the P- (i.e. Borderline Pass) as a Borderline grade [23]. This study employed data that are most suitable for the OBM2, i.e., assessment data that clearly define Borderline as a grade given when the examiner cannot clearly determine whether the examinee performed at a Pass or Fail level.

When considering all responses to a single item given by all students, the OBM is an index of the difficulty of an item (‘Difficulty’). When considering all responses to all items within a station given by a single student, the OBM is then an index of student ability (‘Ability’). The OBM2 is a technique that uses these two OBM indices to make pass/fail decisions for B grades. It works by two OBM indices being calculated for each B grade: an OBM index describing item difficulty (‘Difficulty’) and an OBM index describing student ability (‘Ability’). If Ability ≥ Difficulty then the B grade is reclassified as P grade, otherwise if Ability < Difficulty the B grade is reclassified as an F grade.

Although inspired by Item Response Theory (IRT), the OBM2 is by no means a form of IRT, nor is it an alternative to IRT models. The OBM2 is used only in relation to a particular type of examination consisting of three types of grades: Fail, Borderline and Pass (and above), and its only purpose is facilitating pass/fail decisions when borderline grades are awarded. Nonetheless, the similarity to IRT is that item difficulty and student ability are measured on the same scale, thus they are comparable. The OBM2 is applicable only when items underlie a single construct. Previous studies demonstrated that, to reach a high level of accuracy, items need to be loaded on a single factor and yield at least a moderately acceptable level of reliability (Cronbach’s alpha > .60) [23].

Table 1 demonstrates how the OBM2 is applied. This example is taken from one station in one of the OSCEs conducted at one of UNSW’s clinical examination sites. The OSCE station consists of five assessment criteria (Items 1–5) and there are 15 examinees. For each item, students can be awarded an F, B, P or P+ grade. As described earlier, each grade was converted to a numeric score F (=3), B (=5), P (=7) or P+ (=9) for analysis. This produces a “raw” score. OBM indices (Ability and Difficulty) are calculated for each item and each student when applicable (if no B grade was obtained, no OBM is calculated). Then for each B grade, a comparison between Ability and Difficulty is made as described above. The arrows on the right hand-side of the 5’s (numerical mark under each item) indicate whether the 5 is modified to 7 (↑) or to 3 (↓). This conversion was made to align with the scoring conversion at UNSW Medicine Program as described above (i.e. *P* = 7 and F = 3). The two right hand columns compare each student’s mean score, before and after the OBM2 was applied. The 5 mark is the cut-score as determined by university and faculty policies, and this cut-score cannot be changed. In this demonstration, students 5 and 13 would have passed the OSCE prior to the application of the OBM2. However, once applied, the OBM2 determined that these students should fail. Item 5 is difficult (OBM index = 0.462, mean score = 6.46) and thus B grades in this item are modified upwards. Item 4 is easy (OBM index = 0.864, mean score = 7.00) and thus B grades in this item are modified downwards. The grades in this demonstration yield high internal consistency (Cronbach’s alpha = .888). Readers may scrutinise the table to see how the OBM2 applies across students and items. This table is readily constructible using Excel™, and readers may test it using their own data.

**Table 1:**
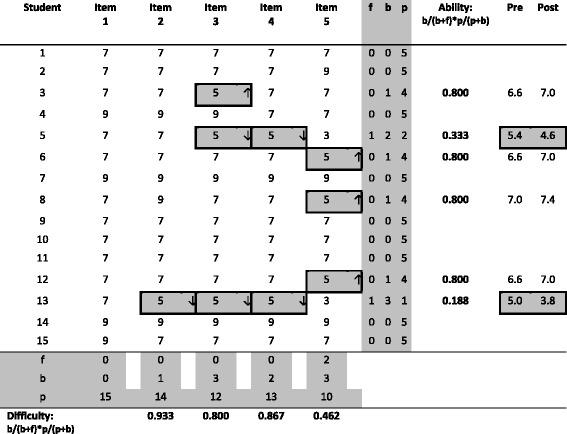
The application of the OBM2 (one station in one site)

The main objectives of this study were to identify the impact of the implementation of the OBM2 in high stakes OSCE on examination results, and to assess the validity and defensibility of the application of OBM2 to a high stakes OSCE in a Medicine program.

The study was approved by the UNSW Human Research Ethics Advisory (HREA) Panel, reference HC15421.

## Method

### Sample

The study population consisted of 259 students in the sixth (final) year of the Medicine program at UNSW. The data are derived from the final OSCE undertaken in the Medicine program in 2016.

### OSCE stations

The OSCE consists of nine stations which are organised by discipline: Medicine (2 stations); Surgery (2 stations); Psychiatry; Emergency Medicine; Obstetrics and Gynaecology (O&G); Primary Care (GP); and Paediatrics. Stations may include a real patient or a surrogate, actor or mannequin as applicable. The examination is conducted across five Clinical Schools. Each session may have a number of choices regarding the actual case presented, but the clinical task, skills assessed, assessment criteria and scoring sheet are identical for a given station across all clinical school sites within each discipline.

### Statistical analysis

Factor analysis using maximum likelihood with oblimin rotation was employed followed by measurement of Cronbach’s alpha for internal consistency to identify whether the a single construct underlined the data within each station [33].

Differences between the mean scores generated from the raw and the modified scores were measured using paired *t*-tests.

Since university policy is that 50% is a pass/fail cut-score, the cut-score for the each station must follow suit. Therefore to pass a station, each student needs to achieve a mean score ≥ 5 (out of a maximum of 10). Students are required to pass the overall examination and the individual disciplines (marks from the OSCE are combined with marks from the written examination and viva). Interclass correlations between the mean scores at each station and the pass/fail decision were calculated for the raw and modified scores (denoted as the ‘traditional’ method and the OBM2 respectively).

Number and proportions of fails per station were calculated across the two methods using the raw scores (henceforth: the traditional method) and the modified scores (OBM2). Bootstrapping was used to estimate 95% confidence intervals for these proportions [34].

## Results

When introduced several months prior to the examination taking place, the change from P- to B grades was welcomed by students and examiners alike as easier to understand, thus likely to be more reliable and fairer. No complaints from examiners or students were raised across all clinical examination sites. In calculating grades after the exam, replacement of the P- with the B grade made the marking schedule and algorithm simpler and more coherent for exam administrators.

The first analysis tested whether a single construct underlined all items within each station. Table 2 demonstrates that the responses to assessment criteria (items) within each station were loaded on a single factor with a high level of reliability, which confirms the suitability of these data for the OBM2.

**Table 2:** Measurement of unidimensionality (Factor analysis and Cronbach’s alpha)

Station	No. of factors	Variance explained	Reliability
Med.A	1	50.6	0.828
Med.B	1	50.3	0.848
Surg.A	1	47.9	0.816
Surg.B	1	52.6	0.853
Psych	1	47.7	0.813
E.Med	1	47.1	0.834
O&G	1	40.5	0.796
GP	1	50.7	0.836
Paed	1	45.8	0.803

A comparison of the final marks awarded to students per station by each method (Table 3) demonstrates that no meaningful differences were observed between mean scores across methods and stations. Even when differences were statistically significant (measured by paired *t*-test) the magnitudes were practically negligible.

**Table 3:** Comparison of mean marks per station per method

Station	Method	Mean	Std. deviation	Std. error mean	Mean diff	95% CI		Sig
						Lower	Upper	
Med.A	(P-)	7.22	1.01	0.062	0.07	0.02	0.12	0.004
	(B)	7.15	1.18	0.073				
Med.B	(P-)	7.14	1.06	0.066	0.04	0.00	0.09	0.073
	(B)	7.10	1.23	0.076				
Surg.A	(P-)	7.20	0.91	0.057	−0.01	−0.05	0.04	0.724
	(B)	7.20	1.03	0.064				
Surg.B	(P-)	7.24	0.88	0.055	0.01	−0.03	0.06	0.590
	(B)	7.23	1.02	0.064				
Psych	(P-)	7.21	1.06	0.066	0.08	0.04	0.13	0.000
	(B)	7.13	1.24	0.077				
O&G	(P-)	7.24	1.13	0.070	0.04	−0.01	0.08	0.085
	(B)	7.20	1.30	0.081				
P.Care	(P-)	6.98	1.13	0.070	0.01	−0.04	0.06	0.732
	(B)	6.98	1.30	0.081				
Paed	(P-)	7.38	0.91	0.057	0.06	0.01	0.10	0.009
	(B)	7.32	1.08	0.067				
E.Med	(P-)	7.32	1.01	0.063	0.03	−0.01	0.07	0.179
	(B)	7.30	1.16	0.072				

*N* = 259

(B) Marks calculated using OBM2

(P-) Marks calculated using the ‘traditional method’

The next analysis compared the efficacy of the pass/fail decisions across the OBM2 with the traditional method while duly applying university policy, i.e. a mean score ≥ 50% grants a pass and <50% results in a fail. Pass/fail decisions were determined in two ways. First, mean marks were calculated using the raw marks as given by the examiners without any modifications (traditional method). Then, the OBM2 was applied and all the B grades (=5) were reclassified as either F = 3 or *P* = 7 and pass/fail decisions were determined using the means of the modified grades (OBM2). Interclass correlations between observed (raw) numerical scores and the pass/fail decision made by the two methods (traditional and OBM2) were calculated and compared (Table 4). The results clearly demonstrate that the OBM2 pass/fail decisions yielded higher correlations with the observed marks compared to the traditional method (except for the Emergency Medicine station where there was no difference). Note that the R^2^ yielded by the OBM2 is in most cases almost 50% higher than the R^2^ yielded by the traditional method (Table 4).

**Table 4:** Interclass correlations between raw mark and pass fail decision by decision method

Station	Traditional method	R^2^	(OBM2)	R^2^
Med.A	0.383	14.7%	0.497	24.7%
Med.B	0.400	16.0%	0.563	31.7%
Surg.A	0.372	13.8%	0.492	24.2%
Surg.B	0.313	9.8%	0.450	20.3%
Psych	0.446	19.9%	0.660	43.6%
O&G	0.588	34.6%	0.690	47.6%
P.Care	0.378	14.3%	0.546	29.8%
Paed	0.308	9.5%	0.428	18.3%
E.Med	0.426	18.1%	0.426	18.1%

*P* < 01 for all correlations; *N* = 259

(B) Marks calculated using OBM

(P-) Marks calculated using the traditional method

The final analysis compares failure rates per station in the OSCE across the two methods. The implementation of the OBM2 increased the failure rate by 2–3 fold across all stations, except for the Emergency Medicine station (Table 5).

**Table 5:** Failure rate by station by method

	Traditional method				OBM2			
Station	*n*	%	Lo	Hi	*n*	%	Lo	Hi
Med.A	5	1.93%	0.39%	3.86%	14	5.41%	2.70%	8.49%
Med.B	6	2.32%	0.39%	4.25%	19	7.34%	4.25%	10.81%
Surg.A	4	1.54%	0.39%	3.09%	11	4.25%	1.93%	6.95%
Surg.B	2	0.77%	0.00%	1.93%	11	4.25%	1.93%	6.95%
Psych	6	2.32%	0.77%	4.25%	17	6.56%	3.86%	9.65%
O&G	11	4.25%	1.93%	6.56%	22	8.49%	5.41%	11.97%
P.Care	7	2.70%	0.78%	5.01%	22	8.49%	5.02%	11.97%
Paed	2	0.77%	0.00%	1.93%	8	3.09%	1.16%	5.41%
E.Med	5	1.93%	0.39%	3.86%	5	1.93%	0.39%	3.86%

(Lo, Hi indication for 95% CI’s calculated by using bootstrapping); *N* = 259

## Discussion

The main objectives of this study were to identify the impact of the OBM2 on OSCE examination results and to assess the validity and defensibility of applying OBM2 to high-stakes examinations in Medicine programs.

In this study, we compared two methods for determining pass/fail scores for OSCE stations: (1) The traditional method, which used the indeterminate Borderline Pass grades (i.e. P- = 5) to calculate a mean score for the station [27]; and (2) the OBM2 [23], which reclassified indeterminate Borderline grades as either Pass or Fail based on the distribution of all grades across all students and stations.

The current study differs significantly from all previous studies investigating the OBM in that this is the first report of outcomes of an OSCE purposely designed to utilise the OBM2. Previous studies either used simulated data to demonstrate the utility of the OBM [22] or used observed data that had not been generated with the OBM in mind, and were not purposefully designed for it [21, 23, 24]. Moreover, this is the first time that results using the OBM2 have had a practical impact on student outcomes. All previous studies investigated hypothetical outcomes had the OBM been implemented. The following discussion addresses the results in that context.

The main findings of this study are: (1) the implementation of the OBM2 did not have any adverse impact on students’ *mean scores* in each of the OSCE stations; (2) compared with the traditional method, the OBM2 pass/fail decisions yielded higher correlations with the original grades awarded by the examiners; and (3) the OBM2 increased the fail rate per station in eight out of nine OSCE stations.

The main question is, therefore, whether the implementation of the OBM2 has been successful and whether it is a method with potential for widespread use for clinical examinations?

To answer that critical question there is a need to consider the following: (1) evidence supporting the validity and defensibility of the OBM2; (2) the impact of the OBM2 on student outcomes; and (3) the acceptability of the OBM2 process and outcomes to the main stakeholders.

### Evidence for validity and defensibility

Table 2 suggests that all items represented a single construct within each station [35], which means that it is acceptable to utilise all grades of all students within a station to determine whether an indeterminate grade (B) should be reclassified as P or F. This is supported by the AMEE guide no. 49, which strongly supports standard setting methods that “use all the assessment interactions between assessors and candidates, and these interactions are ‘real’” [20].

Table 3 demonstrates that the change made by the OBM2 to student *mean scores* was negligible, even when the differences were statistically significant. This finding suggests that the OBM2 is relatively balanced in terms of decision making. On the other hand, Table 5 demonstrates that despite the slight change in mean scores, the proportion of students who failed at the station level significantly increased across almost all stations. The explanation for this phenomenon lies within Table 4 which clearly demonstrates that the pass/fail decisions had much higher correlations with the reclassified OBM2 scores than with the raw scores prior to the reclassification. This finding provides strong support for the validity of the OBM2, since it demonstrates that without changing the assessment criteria (the assessment criteria for clear pass and clear fail are predetermined and had not changed from previous years), the implementation of the OBM2 strengthened the association between these decisions and the observed marks. Thus, the OBM2 better utilises the borderline grades than the traditional method. Conversely, had these correlations (Table 4) weakened following the implementation of the OBM2, that would have suggested that the OBM2 lacks validity.

The OBM2 has a few more features that enhance its defensibility. First, *it is not a standard setting method*. The OBM2 *does not* change any predetermined standards set by the organisation but rather enhances them. The OBM2 applies the institutional policy for pass/fail decisions (50% in the current study) and is therefore aligned to institutional standards. By utilising this practice, the OBM2 can be deemed ‘objective’, since it is “based on predetermined criteria, using a large number of assessors and generates a wide range of metrics” [20].

At the examination level, the OBM2 uses the competency standards for clear pass and clear fail which are unequivocally acceptable for program leaders and examiners (i.e. clear pass and clear fail). Employing these indisputable standards, the OBM2 utilises information already embedded in all assessment grades to facilitate pass/fail decisions for indeterminate grades. The OBM2 does not require any additional judgement to make these decisions, and thus is less susceptible to further bias [36, 37]. In addition, the OBM2 considers measures of item difficulty in pass/fail decisions, i.e. the more difficult the item, the more likely that B grades are to be reclassified as P. This feature is important as it provides some remedy for unavoidable variance in item difficulty. Overall, the results and the theory underlying the OBM2 strongly support the validity of the OBM2 as a method for reclassifying borderline grades as either pass or fail [38, 39].

### Impact of the OBM2 on student outcomes and acceptability of the OBM by main stakeholders

The impact of the implementation of the OBM2 on student outcomes provides evidence that the failure rate at the level of each station increased significantly (Table 5). However, the OBM2 *did not* impact any grade given to a student when the level of performance was clearly pass or clearly fail. This means that no student who performed well was negatively impacted by the OBM2. Moreover, students who gained a pass mark in at least three out of the five assessment criteria (items) in a station could not have failed (minimum score for that would be (7 + 7 + 7 + 3 + 3)/5 = 5.4, i.e. Pass). The traditional method permitted a pass for a station with only two clear pass marks out of five. Therefore, employing the OBM2 method has provided a more trustworthy measure of clinical competence, which is what medical schools, healthcare providers and their patients require [38].

From the student perspective, such outcomes might have raised concerns: the students want to pass the OSCE and any rise in failure rate may not be welcomed. However, our discussions with student representatives revealed that although a few concerns regarding the change in grading system had been raised *prior* to the examination, *after* the examination the general response by students was that the Borderline grade “felt fairer”, resulted in a clear grade and took station difficulty into account. It is also noted that the implementation of the OBM2 in the OSCE kept the overall failure rate for the examination at 5.5%, which is lower than typical OSCE results reported in the literature (for example see: [12, 40, 41]).

A convenience sample survey of experienced examiners from different disciplines reported the Borderline grade to be clear, and was preferred to the previous (‘traditional’) grading system. They reported that this change did not alter the frequency of P or F grades appreciably. This supports examiners’ claims that the Borderline grade had not influenced their passing standard. Furthermore, after reviewing the implementation and the results of the OBM2, the Faculty’s Curriculum Development Committee has decided that the OBM2 will be used for all future clinical examinations.

## Conclusions

The OBM2 provides an effective, feasible and defensible solution for utilisation of borderline grades generated in clinical examinations. The current study demonstrated that the reclassification of borderline grades was valid, and that the outcomes were readily defensible. The impact on students’ examination outcomes was acceptable, and all major stakeholders expressed strong support for using this method in the future. Given that the OBM2 was implemented in a high-stakes clinical examination adds weight to its acceptability.

Further research may establish the generalisability of the OBM2, as well as its limitations.

## Abbreviations

OBM: Objective Borderline Method
OSCE: Objective Structured Clinical Examination
